# The silibinin-loaded Zein-β cyclodextrin nano-carriers (SZBC-NCs) as a novel selective cancer cell drug delivery system in HT-29 cell line

**DOI:** 10.1038/s41598-024-65881-w

**Published:** 2024-06-26

**Authors:** Muslim Abdulrazzaq A. al Alabdullah, Mohammad Taghi Goodarzi, Masoud Homayouni Tabrizi

**Affiliations:** 1 Department of Biology, Science and Research Branch, Islamic Azad University, Tehran, Iran; 2 Department of Biochemistry, Shahrood Branch, Islamic Azad University, Shahrood, Iran; 3 Department of Biology, Mashhad Branch, Islamic Azad University, Mashhad, Iran

**Keywords:** Silibinin-loaded Zein-β cyclodextrin nano-Carriers (SZBC-NCs), Antioxidant activity, Selective cytotoxic potential, Cancer cell, Related apoptotic activity, Biochemistry, Chemical modification

## Abstract

Entrapping phytochemical bioactive compounds into nano-structured biocompatible polymers has been successfully utilized for improving cancer treatment efficiency. Silibinin is a potent compound that shows promising anticancer properties. In the present study, the Zein-β-cyclodextrin complex was used to encapsulate silibinin and evaluate the induced cell death type and cytotoxic impacts on human cancer cells. The silibinin-loaded Zein-β cyclodextrin nano-carriers (SZBC-NCs) were synthesized utilizing a gradual ultrasound-mediated homogenization technique and characterized by Zeta potential, DLS, FESEM, and FTIR analysis. The SZBC-NCs’ antioxidant activity was studied by conducting ABTS and DPPH radical scavenging assays. Finally, the SZBC-NCs selective toxicity and cellular death induction mechanism were studied on the HT-29 and AGS cancer cells by measuring the cell survival and apoptotic gene (Caspase 3, 9), respectively, which were verified by conducting the DAPI staining analysis. The negatively charged (− 27.47 mV) nanoparticles (286.55 nm) showed significant ABTS and DPPH radical scavenging activity. Moreover, the remarkable decrease in the IC50 concentrations of the SZBC-NCs among the HT-29 and AGS cancer cell lines exhibited their selective cytotoxic potential. Also, the overexpressed apoptotic (Caspases 3 and 9) and down-regulated necrotic (NFKB) gene expressions following the SZBC-NCs treatment doses indicated the apoptotic activity of SZBC-NCs, which were verified by the increased apoptotic morphology of the DAPI-stained HT-29 cancer cells. The antioxidant and colon cancer cell-related apoptotic activity of the SZBC-NCs make it an appropriate anti-colon cancer nano delivery system. Therefore, they can potentially be used as a safe efficient colon cancer treatment strategy. However, further in vivo experiments including animal cancer models have to be studied.

## Introduction

Due to the complex biochemical progressive physiology of the cancer cells, cancer diagnosing and treatment strategies are still not sufficient to overcome its progression and lethal clinical symptoms. Stomach cancer is the 4th cancer death-leading cause worldwide in 2020. Despite the current achievements in diagnosis and treatment strategies for gastric cancer treatment, its survival remains low, which confirms more attention on novel targeted and safe anticancer compounds^1^. Also, in 2019 colorectal cancer was reported as the third cancer death-leading cause worldwide. The inappropriate lifestyle including alcohol consumption, smoking, sedentary behavior, and physical inactivity the incident cases of colon cancer are rapidly growing especially in low-income and middle-income countries^2^.

Given the inadequate treatment outcomes and severe side effects associated with existing cancer chemotherapy approaches, there is a need to concentrate on developing targeted anticancer substances. These substances should specifically address the survival mechanisms of cancer cells, such as altered cell cycle checkpoints, in order to effectively control the advancement and multiplication of cancerous cells^3,4^.

High intrinsic ROS levels in cancer cells result in persistent oxidative stress, fostering numerous genomic alterations. These enhance the cancer cells’ proliferation and survival abilities under chronic, elevated intrinsic and extrinsic ROS. Eventually, this disregard for cell cycle rules promotes cancer growth and metastasis^4^.

One of the main cancer cell survival strategies is to suppress the ROS-mediated apoptosis response by down-regulating the apoptotic gene effectors (Caspase-3 and Caspase-9)^5,6^ and up-regulating the antiapoptotic inducers such as Nf-KB protein^7^. Therefore, investigating the anticancer compounds targeting the cancer survival effectors requirements has the potential to efficiently arrest cancer development. On the other hand, designing the appropriate drug delivery systems not only improves drug accessibility but also increases the selective cellular uptake chance, which has been approved for the recently produced nano-drug delivery systems (NDDS)^8^.

Several types of amphipathic bio-compatible polymers have been used to produce NDDS such as chitosan, PLGA, Zein, and Cyclodextrin derivatives^9–11^. The NDDS are unable to transport their bioactive cargo to the desired target cells and improve the drugs’ solubility, chemical activity, and cellular uptake process^12,13^.

Considering the several types of colon and gastric cancer chemotherapy side effects and lethal side effects, the researchers have focused on developing safe natural phytochemical anticancer compounds such as curcumin, crocin, urolithins, hesperidin, and silibinin to decrease the risk of lethal undesired side effects during the drug administration^14–19^.

Herein, Silibinin, a major silymarin phytochemical extracted from the Silybum marianum’s milk was selected due to its powerful anti-inflammatory, antioxidant, and anticancer therapeutic potential^19^. In the current study, silibinin-loaded Zein-β cyclodextrin (BCD) nano-carriers (SZBC-NCs) were designed, produced, and characterized to evaluate their antioxidant and selective apoptotic inductive potential among both the gastric (AGS) and colon (HT-29) cancer cells compared with the human normal (Huvec) cells.

## Materials and methods

### Materials

β-Cyclodextrin (Merck, Germany), 3-(4,5-dimethylthiazol-2-yl)-2,5-diphenyltetrazolium bromide (MTT), 2,2-Diphenyl-1-picrylhydrazylradical (DPPH), 2,2′-azinobis (3-ethylbenzothiazoline-6-sulfonic acid) (ABTS), and dimethylsulfoxide (DMSO) were purchased from Merck company. The Silibinin and Zein were provided by Golexir (Mashhad, Iran) and Sigma Aldrich (France) companies, respectively. The human colon (HT-29), gastric (AGS), and normal umbilical vein endothelial cells (HUVEC) were provided by the Pastore Institute of Iran.

### SZBC-NCs synthesis

**Scheme 1:**
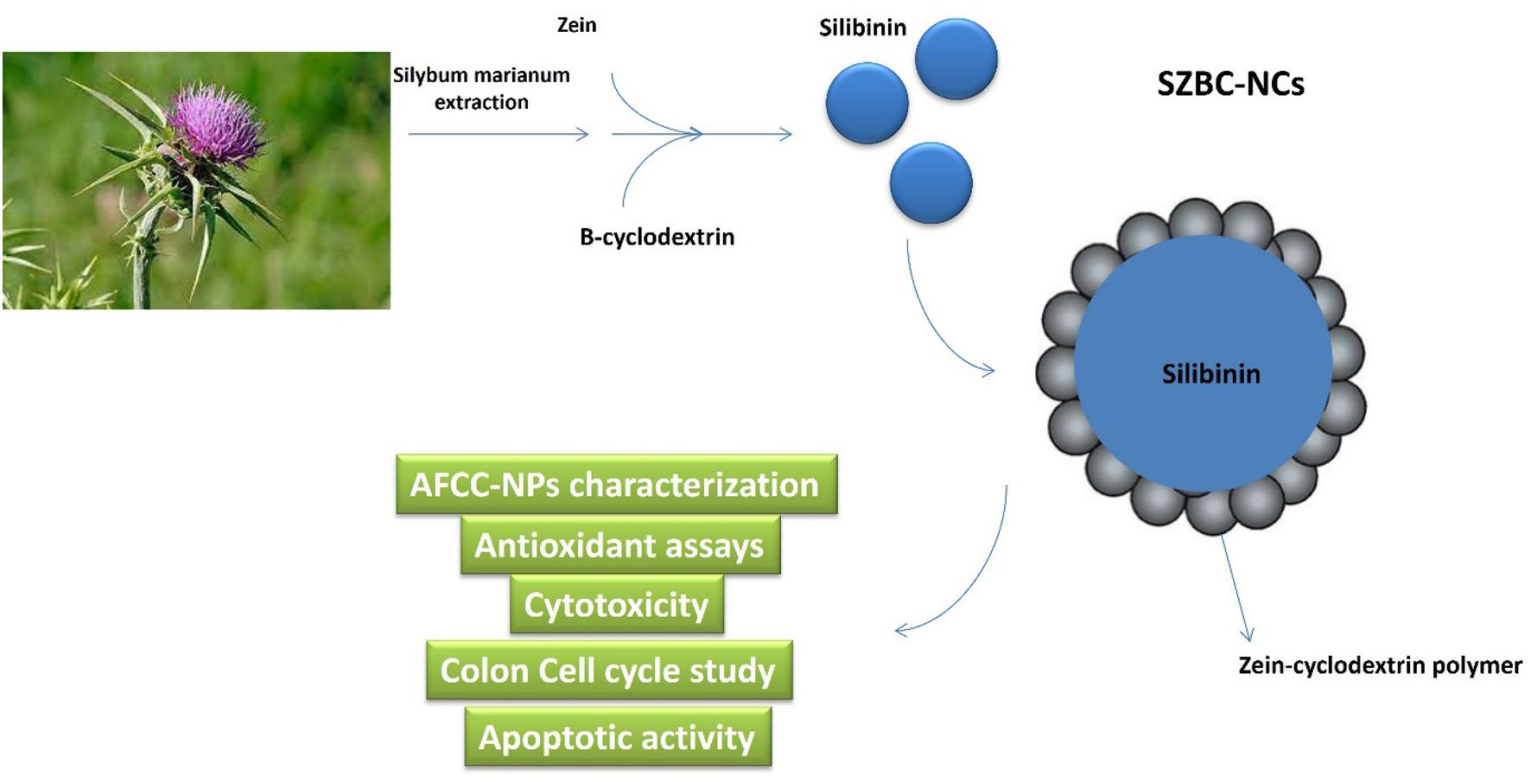
Producing the SZBC-NCs process.

To produce the silibinin-loaded Zein-β cyclodextrin nano-carriers (SZBC-NCs), the nanocarriers’ ingredients were separately dissolved in an appropriate solvent. In this regard, Zein and β-cyclodextrin were dissolved in ethanol (40% V/V) and stirred for 1 h at 50 °C. Then, Silibinin (20 mg) was added to the ethanolic β-cyclodextrin solution. Finally, the cooled solution was dropwise added to the Zein solution under a probe-mediated ultrasound homogenizer device at 300 Watt-power for a 10-min sonication process (8ʹʹ On 2ʹʹ Off). The resulting solution was centrifuged, rinsed with distilled water, and lyophilized applying a − 80 °C—Freez drier (Scheme 1).

### SZBC-NC characterization

The SZBC-NCs’ hydrodynamic and dehydrated size dimensions were performed by dynamic light scattering (DLS) (Zetasizer (nanoparticle SZ-100)) and Field emission scanning electron microscopy (FESEM) techniques, respectively. The DLS analysis was conducted in physiologic pH at 37 °C conditions. Also, the FESEM study was performed by drying a 50-µL sample drop on a clean piece of softened aluminum foil and coating it with a thin ionic gold layer before electron microscopy imaging. The SZBC-NCs chemical composition was characterized by conducting FTIR analysis. To this purpose, 200 mg of potassium bromide (KBr) was added to SZBC-NCs (2 mg) and compressed to a thin disc for scanning the FTIR spectrums in the 4000 to 400 cm^−1^ wavenumbers interval at 4 cm^−1^ resolution. Finally, the nanocarriers’ surface charge was evaluated by applying a Zetasizer device (nanoparticle SZ-100).

### Silibinin loading and releasing efficiency

The Silibinin loading efficiency into the Zein-beta cyclodextrin nanocarriers was determined utilizing a visible-ultraviolet spectrophotometer (Hutch, USA). Briefly, the standard curve of the silibinin was provided recording the absorbance of consent concentrations at 225 nm. The silibinin loading efficiency was estimated by recording the silibinin absorbance before and after the loading process and calculating its concentration considering its provided standard curve. Also, the drug release rate was measured by mixing SZBC-NCs (100 mg) in a phosphate buffer solution and placing it in a pre-activated dialysis bag, which was suspended in a beaker of phosphate buffer. Then, the dialyzed silibinin absorbance was recorded at 225 nm after 6, 12, 24, 48, 72, 96, 120, and 144 h of incubation at 37 °C. Finally, the Silibinin release graph was plotted.

### SZBC-NC antioxidant activity: ABTS and DPPH assay

To measure the SZBC-NCs’ antioxidant activity, their radical scavenging ability was measured as previously described^20^. Briefly, the activated ABTS and DPPH solutions were prepared. The ABTS solution (7 mM) was activated by potassium persulfate (2.45 mM) solution at 1:1 proportion, diluted with water (1:1 V/V), and stored for 14 h at 25 °C in dark conditions. Also, the ethanolic DPPH solution was prepared by dissolving 1 mg of DPPH in 17 mL of ethanol. The antioxidant assay was conducted by separately adding 5 µL of different concentrations of SZBC-NCs mixture (62.5, 120, 250, 500, and 1000 µg/mL) to 3.995 mL of both ABTS and DPPH solutions. The final solutions were kept at room temperature for 30 min in dark conditions. The ABTS and DPPH inhibition rates were measured by recording their related absorbance and the samples absorbance was recorded at 734 and 517 nm, respectively^20^. The inhibition rate of ABTS (IRA%) and DPPH (IRD%) was calculated as the following equation:

$$\text{IRA\% or IRD\%}= \left(\frac{\left[A\right]c-[A]s}{\left[A\right]c}\right)\times 100.$$

### MTT assay

Three different cell lines (HT-29, AGS and Huvec) were purchased from the cell bank of Ferdowsi University of Mashhad (Iran). The cancerous (HT-29 and AGS) and normal Huvec cell lines were seeded (5 $\times$ 10^3^ cells/cm^2^) in a complete DMEM cell culture medium and cultured for 24 h at the standard conditions (5% CO_2_, 37 °C, and 95%humidity). The medium was supplemented with FBS (10%) and streptomycin/penicillin (100 mg/mL/100 U/mL). The 24-h cultured cells were seeded (at 5 $\times$ 10^3^ cells/well density) and incubated for 24 h in 96-well plates. Then, the cells were treated with different SZBC-NCs concentrations (7.8, 15.6, 31.2, 62.5, 125, 250, and 500 µg/mL) for 48 h. Then, the MTT (0.5 mg/mL)-containing medium was replaced and incubated for 3 h at 37 °C. To dissolve the produced formazan, DMSO was added to the wells to record the formazan absorbance at 570 nm (Stat fax 2100 plate reader). The cells’ survival was calculated considering the following equation:

$$\text{Cell survival }(\text{\%}) = \left(\frac{\left[A\right]s}{\left[A\right]c}\right)\times 100.$$

### DAPI staining

The 48-h exposed cells with different doses of SZBC-NPs (205, 225, and 255 µg/mL) were fixed with paraformaldehyde (4%) for 9 min. The PBS and triton X-100 (0.1%) solutions were used for the rinsing and permeabilizing process of the cells. Finally, the fixed-permeabilized cells were stained with DAPI (4,6-diamidino-2-phenylindole) for 5 min. The cells were studied by a fluorescent microscope (Olympus IX81 invert fluorescence microscope), which was equipped with an Olympus DP70 camera (Olympus Corp., Tokyo, Japan)^21^.

### Gene expression profile

The 48-h exposed HT-29 cancer cells with different doses of SZBC-NCs (205, 225, and 255 µg/mL) were harvested for the RNA extraction process. The cells’ total RNA was extracted by utilizing an RNA extraction kit (Pars Tous, Iran) and prepared for synthesizing the cDNA libraries applying a cDNA synthesis kit (Pars Tous, Iran). The Caspase 3, Caspase 9, and Nf-KB gene primer sets were designed by Allel ID6 software applying the exon junction method (Table 1). The target genes’ cDNA was amplified by PCR technique (Bio-Rad CFX96). Also, the Q-PCR method was applied to measure the gene expression profiles compared with the control house-keeping gene (GAPDH) applying the 2^−∆∆CT^ method for calculating the target genes’ fold change values. A SYBR green-supplemented PCR master mix (Qiagen, Hilden, Germany) was used for conducting Q-PCR analysis. Finally, a comparative threshold cycle method was conducted to normalize the fold change values.

**Table 1:** The PCR primer sets of target genes.

Gene	F	R
Caspase-3	CTGGACTGTGGCATTGAGAC	ACAAAGCGACTGGATGAACC
Caspase-9	CCAGAGATTCGCAAACCAGAGG	GAGCACCGACATCACCAAATCC
NFK-B	CCTGCTTCTGGAGGGTGATG	GCCGCTATATGCAGAGGTGT
GAPDH	GCAGGGGGGAGCCAAAAGGGT	TGGGTGCCAGTGATGGCATGG

### Statistical analysis

The statistical measurements were conducted by applying SPSS-20 software. The One-way ANOVA statistical analysis was utilized to determine the statistical significance levels of p-values. The less than 0.001, 0.01, and 0.05 were considered as the statistically significance levels expressed as ***, **, and * index.

## Results

### SZBC-NC characterization

**Figure 1:**
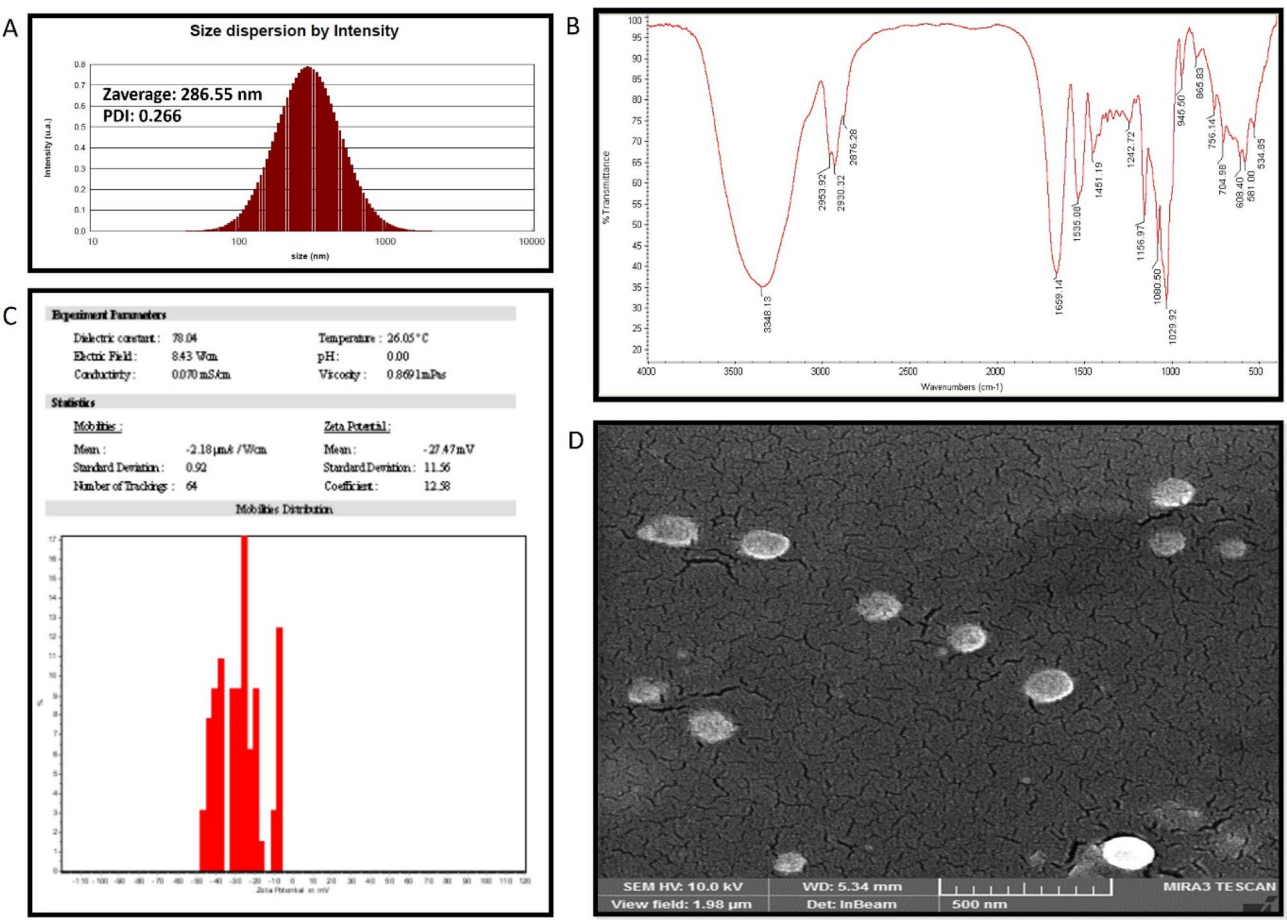
The HG-NPs and SZBC-NCs size characterization. (**A**) The FESEM micrograph of the dehydrated SZBC-NCs size and morphology. (**B,C**) Show the hydrodynamic Z-average size of HG-NPs and SZBC-NCs, respectively. *SZBC-NCs* silibinin-loaded Zein-β cyclodextrin nano-carriers.

The DLS-reported hydrodynamic size of the SZBC-NCs was measured at 286.55 nm (Fig. 1A). Also, the nanocarriers’ Poly-dispersed Index (PDI) was determined at 0.266, which refers to their reliable size mono-dispersion^22^. Moreover, the SZBC-NCs chemical structure was verified by detecting their indicator functional groups and chemical bonds vibrational wavenumbers including the SZBC-NCs’ silibinin, Zein, and β-cyclodextrin, which has been indicated by FTIR spectroscopy in Fig. 1B. The silibinin was detected regarding the plotted appeared spectrum at the vibrational 2876.26 cm^−1^ (OCH_3_), and 1535.08 cm^−1^ (aromatic C=C bending)^23^. The Zein corresponding characteristic bands at amide I, II, and III were detected at 1659 cm^−1^ (stretching C=O bond), 1531 cm^−1^ (bending N–H and stretching C–N bonds), and 1242.72 cm^−1^, respectively^24^. Finally, the β-cyclodextrin polymer was verified by finding the peak at 3348.13 cm^−1^ indicating the stretching vibrational O–H groups. Moreover, the vibrational stretching wave numbers in 3200–3400 cm^−1^ interval exhibited the presence of stretching vibrations of the phenolic hydroxyl group (O–H bonds) of β-cyclodextrin^25,26^. The Zein-β-cyclodextrin shielded layer improves the SZBC-NCs aqueous solubility and chemical stability (− 27.47 mV), which prevents the nanocarriers’ aggregation in an aqueous solution (Fig. 1C)^27^. Finally, the spherical dehydrated 110-nm nanocarriers (SZBC-NCs) were presented by the FESEM microscopy analysis (Fig. 1D).

### Silibinin loading and releasing efficiencies

**Figure 2:**
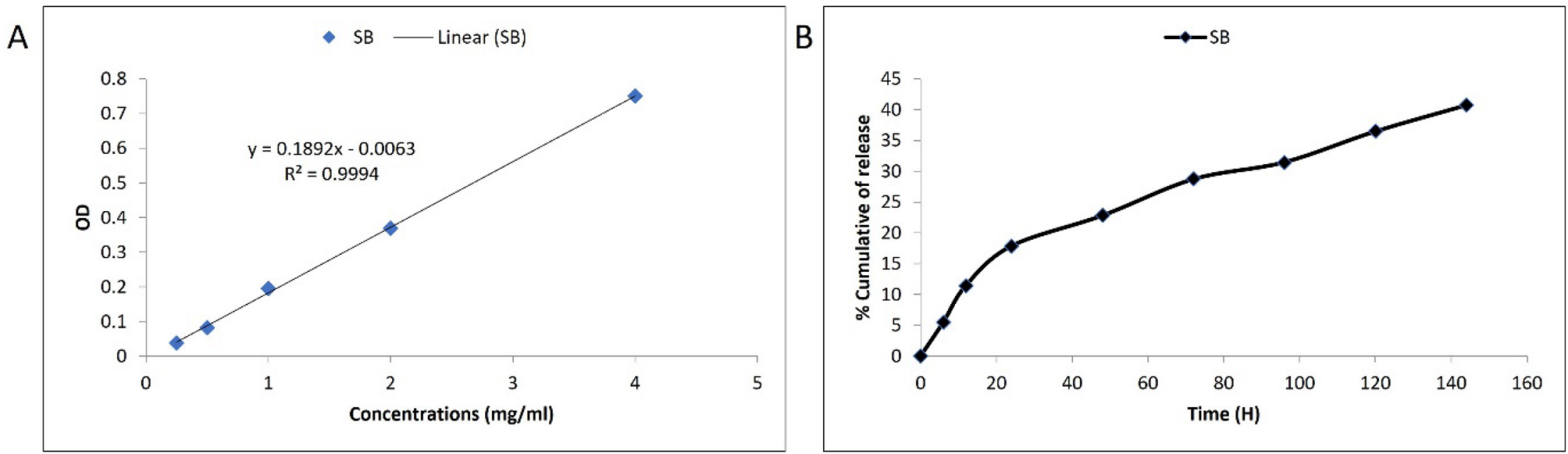
The Silibinin standard (**A**) and releasing curves from the SZBC-NCs (**B**). *SZBC-NCs* Silibinin-loaded Zein-β cyclodextrin nano-carriers.

Considering the silibinin standard curve shown in Fig. 2A, the silibinin loading efficiency (SLE%) at physiologic pH was measured at 87.25%. Also, the silibinin release percentage from the nanocarriers has been calculated at 18% over the first 24 h. The total silibinin releasing efficiency (SRE%) was estimated at over 144 h at 40.75%, which showed its gradual release rate and the appropriate stability of SZBC-NCs (Fig. 2B).

### The SZBC-NC antioxidant activity

**Figure 3:**
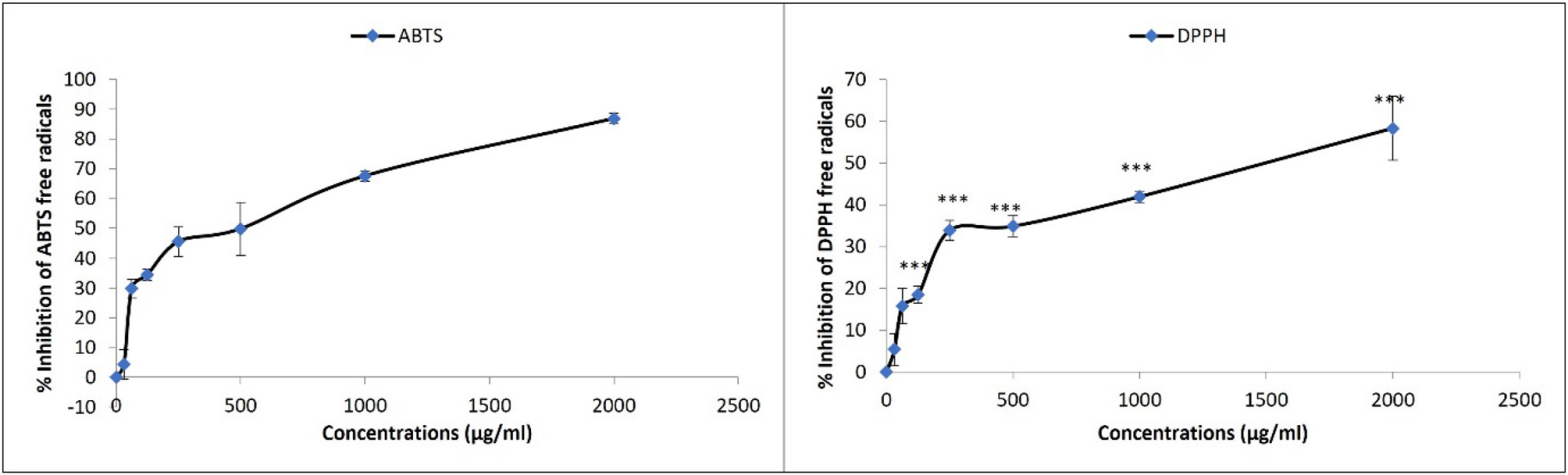
The SZBC-NCs activity in scavenging the ABTS and DPPH free radicals. *SZBC-NCs* Silibinin-loaded Zein-β cyclodextrin nano-carriers.

As shown in Fig. 3 the ABTS and DPPH free radical inhibition rates were illustrated in response to a range of SZBC-NCs concentrations. The results showed a significant positive correlation between the radical inhibition rate and increased nanocarrier concentration. In this regard, the minimum concentrations of SZBC-NCs for conducting 50% ABTS- and DPPH-inhibition were estimated at 500 μg/mL and 1400 μg/mL doses. Moreover, the high dose of SZBC-NCs (2000 μg/mL) exhibited the maximum radical inhibitory rate at 86% and 58% for ABTS and DPPH inhibition, respectively.

### The SZBC-NC cytotoxicity

**Figure 4:**
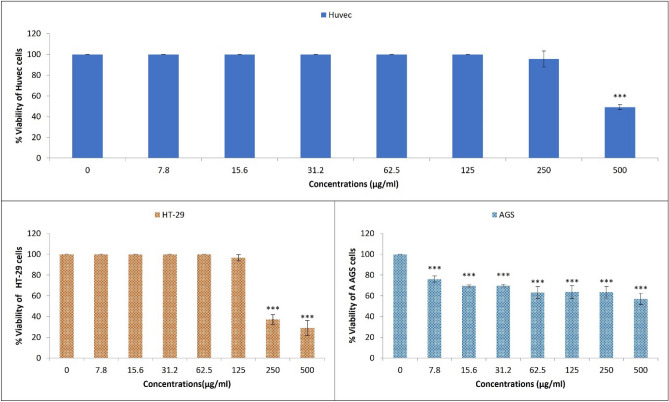
The 48-h treated cell survival in response to the increased SZBC-NCs treatment doses. *SZBC-NCs* Silibinin-loaded Zein-β cyclodextrin nano-carriers.

The SZBC-NCs cytotoxic impact on the human colon and gastric cancer cells are shown in Fig. 4. The results clearly illustrate the significant cell selective cytotoxic impact of SZBC-NCs on the human HT-29 cancer cells compared with other normal Huvec and gastric AGS cancer cell lines. Considering the lowest IC50 concentration of SZBC-NCs (250 μg/mL) in reducing the HT-29 cells’ survival and not significant toxic impact at their greater than 250 μg/mL doses on the other cell lines, the SZBC-NCs exhibited meaningful cancer cell-selective anticancer impact on the treated HT-29 cancer cells.

### The SZBC-NCs apoptotic activity

**Figure 5:**
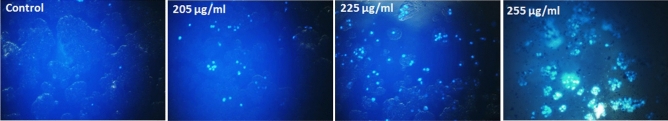
The DAPI staining assay of the treated HT-29 colon cancer cells with different SZBC-NCs treatment concentrations. *SZBC-NCs* Silibinin-loaded Zein-β cyclodextrin nano-carriers.

To clarify the induced cell death type in response to SZBC-NCs treatment concentrations, a DAPI staining analysis was conducted. The cells’ nuclei were stained in the permeabilized apoptotic cells and illustrated with the increased bright nuclei in response to enhanced SZBC-NCs treatment concentrations compared with the not-stained normal cells’ nuclei (Fig. 5). The stained cells revealed their membrane permeabilization, which is considered the apoptotic death hallmark. Therefore, the increased bright-colored bodies following the enhanced SZBC-NCs treatment verify the apoptotic death occurrence. However, further molecular-based analysis is required to evaluate the apoptotic involved gene effectors such as Caspase 3, Caspase 9, and Nf-KB.

**Figure 6:**
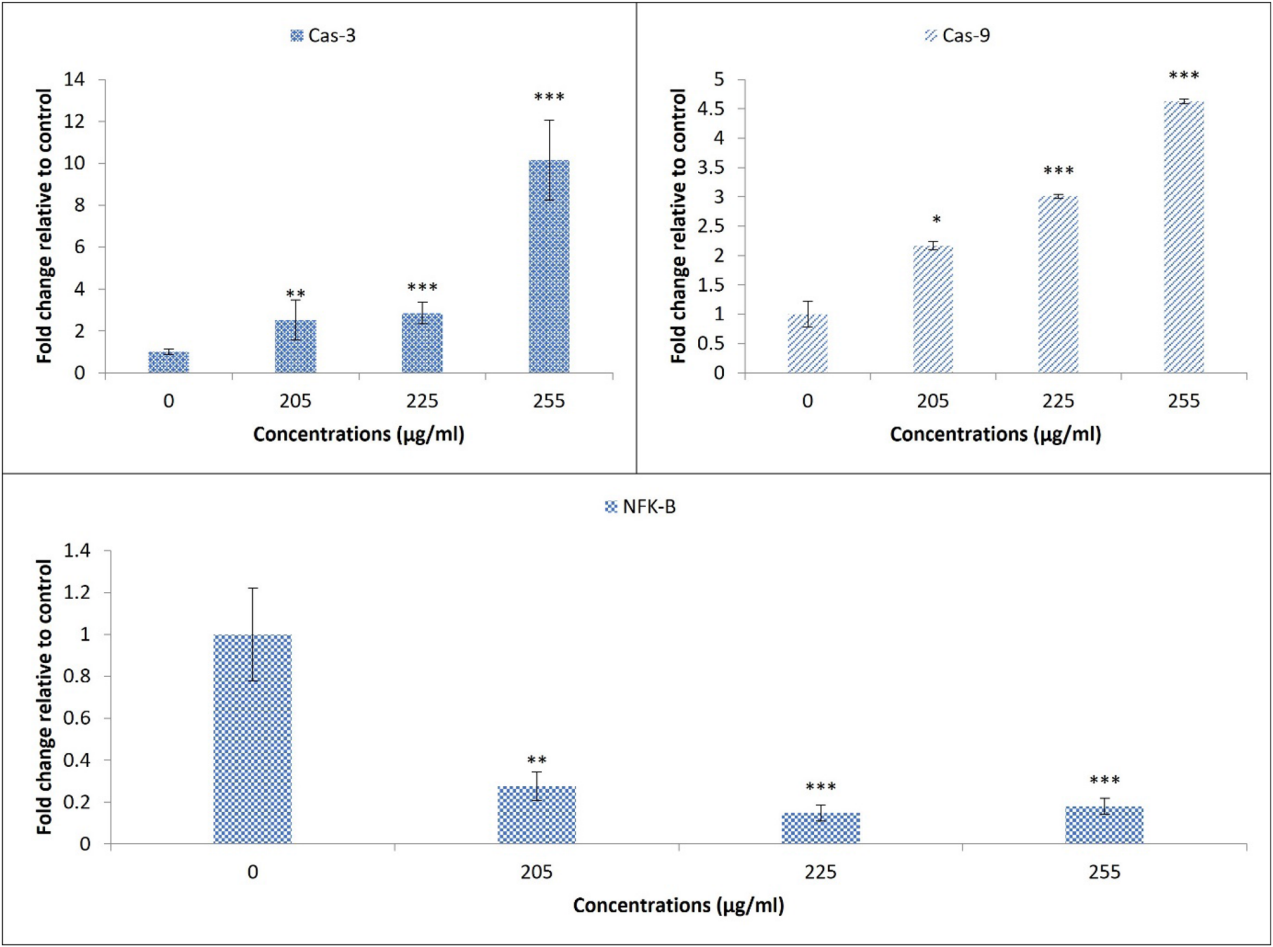
The apoptotic gene expression profile of the treated HT-29 colon cancer cells in response to a range of SZBC-NCs treatment concentrations. *SZBC-NCs* Silibinin-loaded Zein-β cyclodextrin nano-carriers.

In this regard, the expression of the most known apoptotic gene effectors was analyzed in the treated HT-29 colon cancer cells. The significant overexpression of both Caspase 3 and Caspase 9 genes following the increased SZBC-NCs concentrations indicates the simultaneous activation of both the intrinsic and extrinsic apoptosis response in the treated HT-29 cells. This is while the meaningful down-regulation of Nf-KB down-regulates the cancer cell antiapoptotic genes and prevents the drug resistance response in the treated cancer cells^7^ (Fig. 6).

## Discussion

The poor prognosis, delayed diagnosis, and low survival rate of colon and gastric cancer patients are the current challenging problems in the current treatment approaches. Designing functionalized nano drug delivery systems as the appropriate chemotherapy substituent treatment has raised hopes for efficiently suppressing cancer-caused death. In the current study, Silibinin was loaded into the Zein-BCD complex to evaluate its antioxidant and selective apoptotic potential on both human colon (HT-29) and gastric (AGS) cancer cells. The results indicated a significant powerful antioxidant activity of the produced SZBC-NCs and remarkable cell-selective anticancer impact on the human colon HT-29 cancer cells compared with the gastric (AGS) and normal Huvec cell lines.

The less undesired clinical side effects and multiple medicinal applications of the medicinal plants are the two determinant factors making them the only efficient anticancer compound instead of the current chemotherapy drugs. Silibinin as the main phytochemical found in the milk thistle *Silybum marianum* is a nonpolar molecule that poses a wide variety of bioactivities such as anti-inflammatory, anticancer, hepatoprotective, antibiotic, and antioxidant activities^19,28^. However, their lower solubility causes their weak bio-accessibility and shortens their chemical bioactivity in in-vivo aqueous conditions. Nano drug delivery systems have emerged as a promising approach to improve drug solubility, target specificity, and therapeutic efficacy. In this regard several nano drug delivery systems applied for the treatment of colon cancer, such as Polymeric Nanoparticles including poly(lactic-co-glycolic acid) (PLGA), chitosan, or polyethylene glycol (PEG). The primary advantage of polymeric nanoparticles is their ability to protect the encapsulated drug from degradation in the gastrointestinal tract, allowing it to reach the colon intact. Upon reaching the colon, the drug is released due to the change in pH or the action of colon-specific enzymes. This targeted drug delivery enhances the therapeutic efficacy and reduces systemic side effects^29^.

Also, Stimuli-responsive hydrogels are another innovative nano drug delivery system for colon cancer treatment. These hydrogels can respond to specific stimuli, such as pH, temperature, or enzymes, present in the colonic environment. The drug is incorporated within the hydrogel matrix, which remains stable in the stomach and upper intestine. Upon reaching the colon, the hydrogel undergoes a change in its physical properties, such as swelling or degradation, due to the stimuli present. This change triggers the release of the encapsulated drug, ensuring targeted delivery to the tumor site^30^.

Moreover, liposomal drug carriers are spherical vesicles composed of one or more phospholipid bilayers, which can encapsulate both hydrophilic and hydrophobic drugs. These liposomes can protect the drug from degradation and enhance its solubility. For colon cancer treatment, pH-sensitive or enzyme-sensitive liposomes can be designed to release the drug specifically in the colonic environment. This targeted drug release reduces systemic side effects and improves the therapeutic efficacy of the anticancer agents^31^.

In the current study, Zein, the maize endosperm primary protein was selected due to its potential to prolong the biomolecule’s lifespan, its excellent mechanical in producing films, and controllable delivery of bioactive compounds. Moreover, Zein contains several nonpolar and polar amino acid residues, improving its solubility^32^. In other words, Zein acted as the silibinin solubilizer and protector agent in the SZBC-NCs structure.

Also, the cyclodextrins enhance solubility by covering the drugs’ nonpolar groups with its multiple hydroxyl groups^33^ and improve drugs’ bioavailability by reducing their hydrophobicity and thus rectal absorption^34^. In addition, cyclodextrins increase the chemical stability of drugs by entrapping them into their cavity and protecting them^35^.

In the current study, to enhance the SZBC-NCs’ bio-compatibility, stability, and bioactivity, the Zein-BCD complex was used as the nanocarrier structure.

The SZBC-NCs exhibited anticancer activities targeting the cancer cell survival strategies. The weak antioxidant potential of the SZBC-NCs at their IC50 concentrations in HT-29 cancer cells and their powerful radical scavenging activity at higher toxic doses improved their toxicity in cancer cells and protected normal cells against oxidative stresses, respectively.

Herein, the synthesized SZBC-NCs significantly induced a selective cytotoxic impact on the human colon HT-29 cancer cells compared with the normal Huvec and cancerous AGS cell lines. The SZBC-NCs’ selective cytotoxicity may be due to the cancer cell membrane zeta potential alteration compared with other cells’ membrane charge. In this regard, it has been analyzed that cancer cells’ membrane zeta potential (about − 15 mV) is slightly more positive than normal cells (− 25 to − 30 mV)^36^.

## Conclusion

The produced SZBC-NCs simultaneously exhibited weak (low doses) and powerful (high doses) antioxidant activity to improve their toxicity in cancerous cells and protect normal cells against acute oxidative storms, respectively. Also, their cell selective apoptotic impact on human HT-29 colon cancer cells makes them an appropriate selective colon cancer treatment compound. However, the SZBC-NCs’ selective anticancer properties have to be analyzed in further in-vitro and in-vivo studies.

## Data Availability

The datasets used and/or analysed during the current study available from the corresponding author on reasonable request.

## References

[1] Morgan E (2022). The current and future incidence and mortality of gastric cancer in 185 countries, 2020–40: A population-based modelling study. EClinicalMedicine.

[2] Sharma R (2022). Global, regional, and national burden of colorectal cancer and its risk factors, 1990–2019: A systematic analysis for the Global Burden of Disease Study 2019. Lancet Gastroenterol. Hepatol..

[3] Peters JM, Gonzalez FJ (2018). The evolution of carcinogenesis. Toxicol. Sci..

[4] Hashemy SI, Seyedi SMR, Hashemy SI, Seyedi SMR (2021). ROS impacts on cell cycle checkpoint signaling in carcinogenesis. Handbook of Oxidative Stress in Cancer: Mechanistic Aspects.

[5] Devarajan E (2002). Down-regulation of caspase 3 in breast cancer: A possible mechanism for chemoresistance. Oncogene.

[6] Olsson M, Zhivotovsky B (2011). Caspases and cancer. Cell Death Differ..

[7] Xia Y, Shen S, Verma IM (2014). NF-κB, an active player in human cancers. Cancer Immunol. Res..

[8] Patra JK (2018). Nano based drug delivery systems: Recent developments and future prospects. J. Nanobiotechnol..

[9] Bernkop-Schnürch A, Dünnhaupt S (2012). Chitosan-based drug delivery systems. Eur. J. Pharm. Biopharm..

[10] Campos LADA (2023). Zein nanoparticles for drug delivery: Preparation methods and biological applications. Int. J. Pharm..

[11] Tiwari G, Tiwari R, Rai AK (2010). Cyclodextrins in delivery systems: Applications. J. Pharm. Bioallied Sci..

[12] Chavda VP (2022). Nano-drug delivery systems entrapping natural bioactive compounds for cancer: Recent progress and future challenges. Front. Oncol..

[13] Herdiana Y (2022). Drug release study of the chitosan-based nanoparticles. Heliyon.

[14] Choudhari AS (2020). Phytochemicals in cancer treatment: From preclinical studies to clinical practice. Front. Pharmacol..

[15] Wang C-Z (2022). Effects of saffron and its active constituent crocin on cancer management: A narrative review. Evaluation.

[16] Ghobadi N, Asoodeh A (2023). Co-administration of curcumin with other phytochemicals improves anticancer activity by regulating multiple molecular targets. Phytother. Res..

[17] Aggarwal V (2020). Molecular mechanisms of action of hesperidin in cancer: Recent trends and advancements. Exp. Biol. Med..

[18] Al-Harbi SA (2021). Urolithins: The gut based polyphenol metabolites of ellagitannins in cancer prevention, a review. Front. Nutr..

[19] Cai J-Y (2017). Silibinin protects *Staphylococcus aureus* from UVC-induced bactericide via enhanced generation of reactive oxygen species. RSC Adv..

[20] Li P (2011). Free radical-scavenging capacity, antioxidant activity and phenolic content of *Pouzolzia zeylanica*. J. Serb. Chem. Soc..

[21] Chazotte B (2011). Labeling nuclear DNA using DAPI. Cold Spring Harb. Protoc..

[22] Stetefeld J, McKenna SA, Patel TR (2016). Dynamic light scattering: A practical guide and applications in biomedical sciences. Biophys. Rev..

[23] Patel P (2022). Lung cancer targeting efficiency of Silibinin loaded poly caprolactone/pluronic F68 Inhalable nanoparticles: In vitro and In vivo study. PLoS ONE.

[24] Ali S (2014). Zein/cellulose acetate hybrid nanofibers: Electrospinning and characterization. Macromol. Res..

[25] Wongsa P, Phatikulrungsun P, Prathumthong S (2022). FT-IR characteristics, phenolic profiles and inhibitory potential against digestive enzymes of 25 herbal infusions. Sci. Rep..

[26] Rachmawati H, Edityaningrum CA, Mauludin R (2013). Molecular inclusion complex of curcumin-β-cyclodextrin nanoparticle to enhance curcumin skin permeability from hydrophilic matrix gel. Aaps Pharmscitech.

[27] Chen Z, Wu D (2014). Monodisperse BSA-conjugated zinc oxide nanoparticles based fluorescence sensors for Cu2+ ions. Sens. Actuators B Chem..

[28] Wing Ying Cheung C (2010). Silibinin-a promising new treatment for cancer. Anti-Cancer Agents Med. Chem..

[29] Dang Y, Guan J (2020). Nanoparticle-based drug delivery systems for cancer therapy. Smart Mater. Med..

[30] Hajareh Haghighi F (2023). Peptide-hydrogel nanocomposites for anti-cancer drug delivery. Gels.

[31] Ashrafizadeh M (2022). Stimuli-responsive liposomal nanoformulations in cancer therapy: Pre-clinical & clinical approaches. J. Controlled Release.

[32] Rahman M (2023). Fabrication of zein-based fibrous scaffolds for biomedical applications—A review. Macromol. Mater. Eng..

[33] Archontaki H, Vertzoni M, Athanassiou-Malaki M (2002). Study on the inclusion complexes of bromazepam with β-and β-hydroxypropyl-cyclodextrins. J. Pharm. Biomed. Anal..

[34] Arima H (2001). Comparative studies of the enhancing effects of cyclodextrins on the solubility and oral bioavailability of tacrolimus in rats. J. Pharm. Sci..

[35] Arima H (1998). Enhancing effect of hydroxypropyl-β-cyclodextrin on cutaneous penetration and activation of ethyl 4-biphenylyl acetate in hairless mouse skin. Eur. J. Pharm. Sci..

[36] Baca JM (2022). Cells electric charge analyses define specific properties for cancer cells activity. Bioelectrochemistry.

